# Ante Partum Hæmorrhage

**Published:** 1886-02

**Authors:** J. Cummings

**Affiliations:** Austin, Texas


### ANTE PARTUM HÆMORRHAGE.

*By J. Cummings, M. D., Austin, Texas.*

Read at meeting of Travis County Medical Association, November 7, 1885.
(For Daniel's Texas Medical Journal.)

WHILE I do not find the above name now given in the text books to hemorrhage occurring anterior to labor, yet it suits the subject to which I devote this article, based on some clinical facts that have come under my observation within a recent period. Case 1 was a Mrs. H, of German descent, aged about 35 years, multipara. Was called on the night of the 31st July, 1885, and found on entering room, my patient pale, pulse feeble; features and expression all indicating extreme suffering and loss of blood. I soon learned that a German midwife had been called and that she had abandoned the case, evidently fearful of the result, and told them to send for a doctor. On examination, I found some slight loss of blood, but not sufficient to account for the blanched and low condition of my patient. I waited an hour or two and found still some hæmorrhage, but still not enough to produce the condition referred to. The pains seemed to be doing no good, the os was dilated to about the size of a twenty-five cent piece, and I felt certain that my patient would die of exhaustive internal hemorrhage, if I did not act promptly and deliver the child. I went home but a few blocks distant, and procured my short forceps, and by dilating the os somewhat, succeeded in applying them to the child's head, and while yet in the superior strait, and delivered a dead child. I will state, there had been no movements of child in utero for several hours, and there were no other signs of life. I immediately delivered the afterbirth by manual effort, and then examined the womb over abdomen and found it very much enlarged. I kneaded the womb with my hand, and succeeded in producing firm contraction, and, to my surprise, there was immediately expelled two very large clots of blood—sufficiently large to fill an ordinary sized chamber. I now had a full solution of the cause of the condition of the case. I was still afraid that too much loss of blood had existed, and that she could not recover, but after high fever, evidently of septic origin, she had a sure but slow recovery.

Case 2, was Mrs. B; aged about 30 multipara. Called on the night of the 19th of November, 1885. She had just lost a five months' fœtus, but as the placenta was firmly held by uterine contraction, I had to deliver it by manual effort. High fever existed and had been in progress several days. She had had regular menses up to within one month before I saw her, and then twice—if not oftener—during the last month. I told them that I was afraid that the fever, if it continued, would be serious, as it might be from blood-poisoning, and after prescribing, went home about 6 a.m., but at 12 m., was called again. I visited her and re-examined the womb internally, as there were severe uterine pains. To my astonishment, I found in the womb an elongated clot, three inches by one and a half, firmly attached to fundus of the womb, very hard and consistent, of strongly ammoniacal odor when detached, and presented the appearance of, not putrid decomposition, but of decomposition of the saline matter of the blood. I now had plainly before me facts, not theory to account, not only for the miscarriage, but for the blood-poisoning as well, which was evidently the cause of the fever. After delivering the clot, I was fully apprised of the state of affairs inside, and consequently proceeded to syringe the womb thoroughly with a warm solution of carbolic acid. The detention of this clot did not leave the womb itself in a healthy condition, as considerable inflammation, both of the womb and adjacent structures followed, and she suffered very much pain, soreness and fever, for more than a week. The intra uterine injection was repeated in person with some immediate relief to considerable uterine pain which followed the succeeding day. I have in vain searched to find a parallel case where all the causes were so manifest, where a blood clot had been concealed behind the fœtus possibly for months, and surely for weeks, producing tendency to, and final miscarriage, and endangering the life of the victim by a slow saline decomposition, because sealed from the air there could be no other kind, producing septicæmia, with its fearful results. Sims feared a small quantity of blood in the abdomen after his operations for ovarian tumors, more than pus.

From the above cases two important lessons may be deduced; in the first case, the importance of immediate instrumental delivery where intra uterine hæmorrhage is suspected before labor, and in the last case, it would certainly be proper, where hemorrhage during pregnancy existed, followed by fever of continued type, to empty the womb of its contents and save the patient of the terrible results of death from septicæmia. Cases similar to Case 1, have not received that attention from the profession that their gravity would demand. Goodell, of Philadelphia, has collected one hundred and six cases. Dr. F. Maxwell. of this county, reported to Travis County Medical Society, one case where the lady died before anything could be done to deliver the child and relieve the patient. Dr. B. E. Hadra, now of Austin, informs me of the case of a lady who died in his practice before relief could be given. I believe in any case where there is sharp ineffectual pains in the pregnant state, whether at full term or not, attended with all the symptoms of internal hemorrhage, whether there is extreme show or not, if the patient is faint from loss of blood, pulse shows feebleness, and increased frequency, with blanched complexion and impending death, then the child should be promptly delivered and the womb emptied of its contents and contraction induce if possible.

[Note—Readers are referred to the November number of this journal for an interesting account of "Hæmorrhage before Delivery," by Prof. E. J. Doering, of Chicago, who points out certain diagnostic signs of great value.—Ed.]

